# Intermittent Voluntary Isometric Contractions Effects on Performance Enhancement and Sticking Region Kinematics in the Bench Press

**DOI:** 10.5114/jhk/161777

**Published:** 2023-04-20

**Authors:** Arkaitz Garbisu-Hualde, Laura Gutierrez, Eneko Fernández-Peña, Jordan Santos-Concejero

**Affiliations:** 1 Department of Physical Education and Sport, University of the Basque Country UPV/EHU, Vitoria-Gasteiz, Spain.

**Keywords:** training, warm up, strength training, athletic performance

## Abstract

During the last years, post-activation performance enhancement (PAPE) has gained notorious popularity due to the capacity to improve the acute rate of force development (RFD) using different strategies with different muscle contraction regimes as conditioning stimuli. The aim of the present study was to analyse the role of a maximal isometric post-activation performance enhancement (PAPE) protocol in performance and its effects on the kinematics of the sticking region. Twenty-one trained participants (age 26.4 ± 5.4 years) underwent two experimental sessions: an experimental session consisting of a single set and a single repetition of the bench press at the 93% of 1RM (which is considered a traditional conditioning activity to induce PAPE) (TRAD) and an isometric experimental session (ISO) consisting of 15 maximal voluntary isometric contractions in the sticking region of the medium grip bench press lasting 1 s with a 1 s rest interval between contractions. Both TRAD and ISO experimental conditions improved performance from post0 to post4, post8, post12 and post16, but only the ISO condition improved performance from the start of the lift to the start of the sticking region from pre to post (p < 0.001), and only the ISO condition improved maximum (p = 0.005) and minimum (p = 0.025) peak velocities. The results of this study suggest that short duration maximal voluntary isometric contractions improve the velocity of the lift prior to the initiation of the sticking region, which ultimately improves the impulse and facilitates the lift.

## Introduction

Post-activation performance enhancement (PAPE) is a phenomenon that acutely improves muscular performance after a conditioning activity (Blazevich and Babault, 2019). PAPE should not be confused with post-activation potentiation (PAP), even if both can be induced by voluntary contractions. Contrary to PAPE, which refers only to performance enhancements, PAP is confirmed via twitch verification tests (Cormier et al., 2022). PAPE is commonly achieved with heavy resistance exercises (the conditioning activity) prior to performing a biomechanically similar movement and its magnitude and duration depend on several factors, including the muscle fiber type, muscle temperature and sarcomere length (Blazevich and Babault, 2019). Due to major sensitivity of type II fibers to calcium concentration, athletes with a higher percentage of type II fibers will benefit more from high intensity PAPE protocols (Blazevich and Babault, 2019). Considering that the fiber type percentage is not fully inherited (Simoneau and Bouchard, 1995) and that training background can determine to a large extent fiber type proportion (Plotkin et al., 2021), interindividual differences in the response magnitude are acceptable.

During the last years, PAPE has gained notorious popularity due to the capacity to improve the acute rate of force development (RFD) using different strategies with different muscle contraction regimes as conditioning stimuli (Gepfert et al., 2019; Krzysztofik et al., 2020; Skurvydas et al., 2019). Various load protocols, such as optimal power loads (Gilbert and Lees, 2005), medium loads (Lowery et al., 2012) and even plyometric exercises (Krzysztofik and Wilk, 2020) have been used. Regarding isometric contractions, current evidence suggests that 15 short, intermittent, and repetitive maximal voluntary contractions (15-MVC) seem to be the most effective isometric strategy to induce PAP (Skurvydas et al., 2019), as changes in the maximal voluntary contraction 10 min after the conditioning activity were highest under that condition. Interestingly, electrically induced twitch torque in 5-s sustained isometric contraction (MVC-5s) condition was higher than in 15-MVC condition (Skurvydas et al., 2019). However, since those results were obtained bypassing the nervous system fatigue with electrical stimulation, we decided to test the protocol that achieved better results in the voluntary contraction test.

Some studies including lower limb exercises, which focused on isometric contractions as potentiation stimuli, have been already performed . Bogdanis et al. (2014) compared the effects of concentric, eccentric and isometric half-squat protocols on countermovement vertical jump performance and they concluded that 3 sets of 3 s of maximal isometric force (with a 1 min rest interval between sets) improved countermovement vertical jump performance by about 3%. Another study (Vargas-Molina et al., 2021) extends those results demonstrating that both concentric (2 sets of 3 repetitions at 75% 1RM) and isometric (2 sets of 4 s with a load equivalent to 75% 1RM) PAPE protocols improved performance in the countermovement jump. On the other hand, Tsolakis et al. (2011) compared the effectiveness of an isometric protocol consisting of 3 sets of 3 s of maximal isometric contractions separated by 15-s rest intervals in the upper and lower body. In this case, upper body performance did not change compared to baseline, and lower body performance decreased showing a negative correlation between leg force and peak leg power.

The sticking region is the weakest region in the range of motion of any lift (Kompf and Arandjelović, 2017). Several strategies have been proposed in the literature to face it, which include additional loading in that specific region using elastic bands or chains, and also increasing the impulse prior to the sticking region (Kompf and Arandjelović, 2016). Based on Lum and Barbosa (2019), isometric strength training can produce strength improvements in joint angles up to 20⁰– 50⁰ away from the joint angles used during isometric training. Therefore, the question arises whether protocols used by Skurvydas et al. (2019) are effective when used with the intention to acutely improve the impulse before and in the sticking region, and whether there are any differences compared to previous protocols (Bogdanis et al., 2014; Tsolakis et al., 2011). In this regard, the barbell bench press, one of the most popular resistance exercises for upper limbs and one of the three main lifts in powerlifting competitions (Gomo and Tillaar, 2016), appears to be an interesting option to answer this question.

Thus, the aims of this study were: i) to determine whether an isometric PAPE protocol is applicable to field conditions for the medium grip bench press, and ii) to observe whether any changes occur in the kinematics of the sticking region due to the PAPE protocol. We hypothesized that i) an isometric protocol would imply higher velocities of the first half of the lift (i.e., improvements seen by Lum and Barbosa (2019) after several weeks extrapolated to a single session, acute force improvements in trained joint angles and nearby), what would make the lift easier due to a higher impulse prior to the sticking region, and ii) this would be reflected in improved performance or a shortened sticking region.

## Methods

### Participants

Twenty-one participants (age 26.4 ± 5.4 years; body mass 79.4 ± 9.7 kg; body height 176.2 ± 6.9 cm; medium grip bench press 1 repetition maximum (1RM) 97.4 ± 19.8 kg; relative strength (1RM/body mass) 1.22 ± 1.9) with at least two years of resistance training experience voluntarily took part in this study. Participants were required to meet the following inclusion criteria: 1) men between the age of 18–40 years; 2) lack of musculoskeletal disorders or injury in the previous 6 months; 3) experienced in resistance training, defined as consistently lifting weights at least 3 times per week for a minimum of 2 years. A total of 20 participants completed the study: one participant dropped out prior to completion due to personal reasons. We did not control for nutrition nor hydration levels, but participants were told not to make any changes in the above during the testing period. Participants were asked to refrain from training 48 h before each testing session and not to take caffeine. All participants performed the three sessions at the same time of the day with at least 48 h of rest between sessions. Written informed consent was obtained from each participant after a thorough explanation of the testing protocol, the possible risks involved, and the right to terminate participation at will. The study was conducted according to the Declaration of Helsinki, and the Institutional Review Board of the University of the Basque Country (UPV/EHU) approved the experimental protocol.

### Procedures

Participants visited the laboratory on three separated occasions. Prior to every experimental session and the 1RM test, participants performed a standardised warm-up protocol, consisting of 5 min of cycling and bench press warm-up sets consisting of 1 set of 12 repetitions with the barbell only, followed by 3 sets of 8, 6 and 3 repetitions with 40%, 60% and 75% 1RM, respectively. The rest interval between warm-up sets was to 2 min. In every session, during the bench press, participants performed the descent with a 2 s tempo, followed by a 1 s pause in the chest with help of a metronome at 60 beats per minute (BPM) to standardise repetitions. Participants were instructed to perform the concentric phase as fast as possible. Bench press grip width was set at 1.4 times biacromial distance as described elsewhere (Larsen et al., 2021).

### 1RM Calculation

During the first visit, participants underwent a direct 1RM test for the bench press. The 1RM was defined as the highest load lifted by participants without any compensatory movement and only if they completed the pause on the chest properly. When an attempt was successful, the next attempt was decided asking the participant and evaluating the reported mean propulsive velocity by the velocity linear transducer (Speed4Lifts, Spain) (Pérez-Castilla et al., 2019). Participants rested for 3 min between attempts. The test finished when participants reported a rate of perceived exertion (RPE) of 10 in the repetitions in the reserve based RPE scale (Helms et al., 2016). If participants failed an attempt, the weight was reduced by 2.5 kg and another attempt was performed after a 3-min rest interval.

### Sticking Region Identification

In addition, each lift during familiarization was recorded from a side view at 300 Hz using an active LED marker on the barbell’s edge and a high-speed video camera (Casio ExilimEX-F1). Video recordings were analysed using kinematic analysis software Kinovea (version 0.8.15), which is valid, precise, and reliable (Puig-Divi et al., 2019). Data exported from Kinovea to Excel (version 16.16.27) were filtered (Butterworth low pass filter at 5 Hz) and then used to determine where the sticking region was, defined as the region of the lift between the first peak (V_max peak_) in velocity and the first minimum after the peak (V_min peak_) (Escamilla et al., 2000). This region varies inter-individually due to differences in the anatomical cross-sectional area of the muscle, force-length relationship, force-velocity relationship, fatigue, motor unit recruitment, fiber type and biomechanical factors that affect torque development (Kompf and Arandjelović, 2016).

Once the sticking region was detected, the height of the barbell at this region was calculated. Since the sticking region is not a particular point, but a range of motion of the lift, to ensure that the isometric contraction affected the sticking region, the protocol was performed in the middle of this region, as isometric contractions had been demonstrated to strengthen 20º–50º away from the adopted joint angles (Lum and Barbosa, 2019).

### Conditioning Activities

Measurements of the experimental conditions lasted 45 min and were scheduled one week after the first visit to the laboratory. The study followed a within-subject design, where each participant was his own control. In this way, in both experimental sessions, participants performed a pre-conditioning lift (control lift), a conditioning activity and several post-conditioning lifts. Thus, on the second day, participants were randomly assigned to one of the following two experimental conditions: an isometric contraction conditioning protocol (ISO) or a traditional conditioning protocol (TRAD). Volume was not matched between conditioning activities. On the third session, participants changed experimental conditions. After completing the conditioning protocol (ISO or TRAD), participants were asked for the RPE.

Each experimental session consisted of a standardised warm-up protocol followed by a 3-min rest interval and a pre-conditioning lift (control lift), which consisted of 1 set of 1 repetition of the bench press at 85% 1RM. After the pre-conditioning lift, participants rested for 3 min, and then they proceeded with the conditioning activity to which they were randomly assigned.

The ISO conditioning activity consisted of 15 maximal voluntary isometric contractions (MVIC) of 1 s with a 1-s rest interval between contractions (Skurvydas et al., 2019) at their sticking region as previously described. Participants were encouraged to exert force as fast as possible. Isometric contractions were performed by fixing the barbell of a Smith machine at the appropriate height (using a 11 mm diameter rock climbing rope) to match the middle of the sticking region height. The TRAD conditioning activity consisted of 1 set of 1 repetition with 93% of their estimated 1RM of the familiarisation day (Garbisu-Hualde and Santos-Concejero, 2021).

Post-conditioning measurements (1 set of 1 repetition of the bench press at 85% 1RM) were recorded 0, 4, 8, 12 and 16 minutes later (post0, post4, post8, post12 and post16) (Lowery et al., 2012; Mina et al., 2019) using the same velocity linear transducer. Participants were instructed to lift the barbell as fast as possible during the ascending phase of the movement.

If participants improved performance from pre-conditioning to any of the post-conditioning lifts, they were chosen for further analysis of the sticking region kinematics. This distinction between responders and non-responders to the conditioning activity was based on the calculated smallest meaningful difference. When the difference in the best post-conditioning lift and the pre-conditioning lift was higher than the smallest meaningful difference, participants were considered responders and chosen for analysis of the sticking region.

### Statistical Analyses

Data were screened for normality of distribution using the Shapiro-Wilk test. Two-way ANOVA with repeated measures (lift **×** time) was used to determine if any of the post-conditioning lifts improved performance under each experimental condition and to compare same time points across experimental conditions (ISO vs. TRAD). The magnitude of differences of effect sizes (ES) was calculated using Cohen’s *d* (Cohen, 1988) and interpreted as small (>0.2 and <0.6), moderate (≥0.6 and <1.2) and large (≥1.2 and <2) or very large (≥2) according to Hopkins et al. (2009). All statistical analyses were performed using Prism 9 for Mac. Significance for all analyses was set at *p* < 0.05. 95% confidence intervals were reported as 95% [Lower limit, Upper limit]. Additionally, for those participants for whom the conditioning activity improved performance (responders), the velocity until the first peak was measured and compared using one-way ANOVA with repeated measures (lift **×** time), comparing pre-conditioning velocity, post0 velocity and velocity of the fastest time point. To select those responders, we calculated the smallest meaningful difference following the formula below:


$$\sqrt{\frac{2\;\cdot \;\sigma^{\text{2}}}{n}}\cdot {\text{ (z}}_{\text{1-}\beta }{\text{+z}}_{\text{1-}\frac{\alpha }{2}}\text{)}$$

The reported results by the formula were smaller (e.g., measured smallest meaningful difference = 0.005 m•s^-1^) than values reported by the used velocity linear transducer (e.g., 0.23 m•s^-1^). Thus, if any participant improved performance in any post-conditioning lift (e.g., from 0.23 m•s^-1^ to 0.24 m•s^-1^), the difference must have been higher than the calculated smallest meaningful difference. We also performed a Fisher´s exact test to compare if there was a significant difference between the number of responders and non-responders to the conditioning activity in ISO and TRAD groups.

## Results

### Kinematics of the Sticking Region

To analyse changes in the kinematics of the sticking region, participants who improved performance in any time point (i.e., responders) were analysed, even if statistical significance was not reached. The Fisher´s exact test reported a significant difference between the number of responders and non-responders (*p* = 0.001). Thus, seven subjects were analysed for the TRAD condition and 17 for the ISO condition.

When comparing mean velocity from the start of the ascending phase until the first maximum peak in velocity (pre-sticking region), no differences were found in the TRAD experimental condition for responders (*n* = 7; 33.33% of participants) (*p* = 0.229; 95% CI [−0.1545, 0.035]) (Figure 2A).

**Figure 1:**
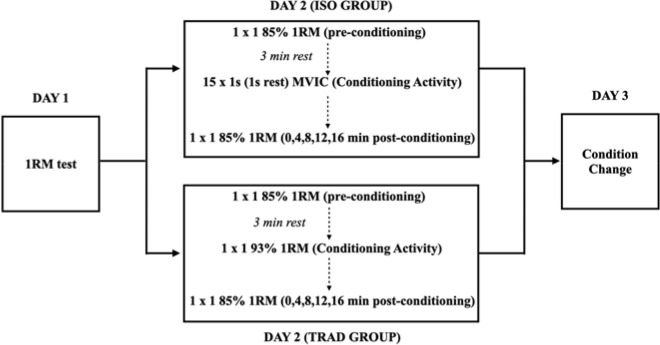
. Scheme of the followed investigation procedure.

**Figure 2:**
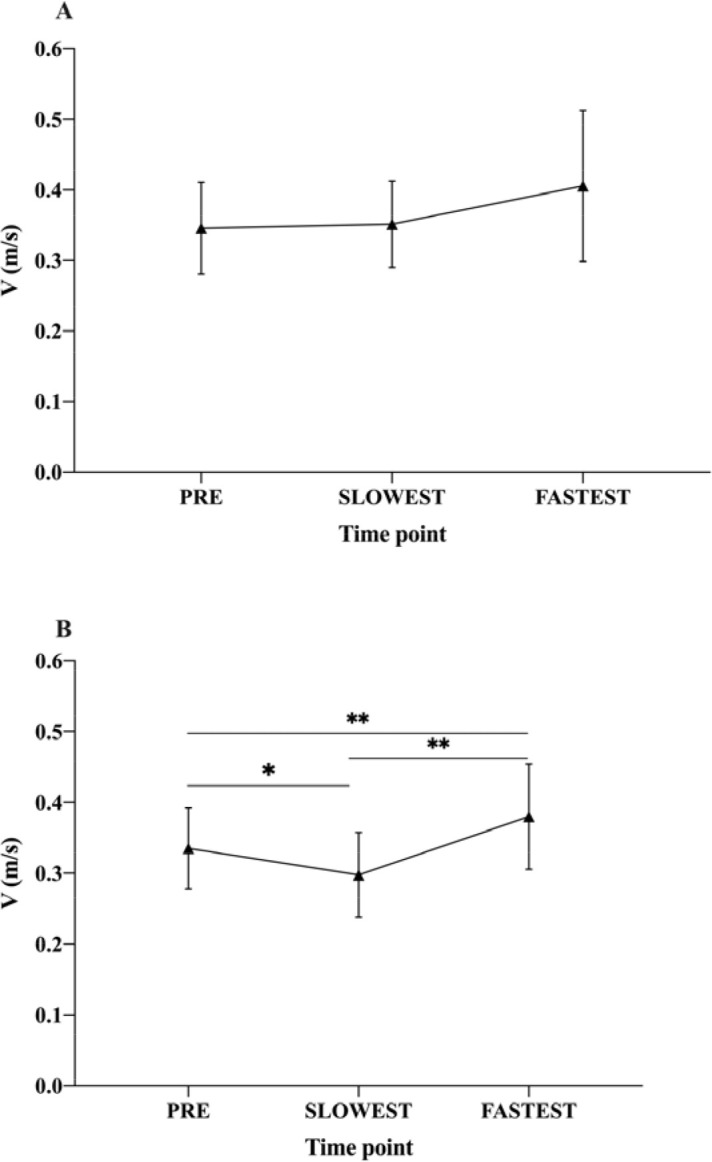
Lifting velocities pre-conditioning and in the slowest and fastest post-conditioning activity time points. (A) TRAD post-activation performance enhancement experimental session. (B) ISO post-activation performance enhancement experimental session. * p < 0.05; ** p < 0.001

In contrast, when comparing the velocity from the start of the ascending phase until the first maximum peak in velocity under the ISO experimental condition in the same time points, we found that for responders (*n* = 17; 85% of participants), the first maximum peak in velocity was higher in the fastest time point (i.e., higher velocities were achieved prior to the initiation of the sticking region) (*p* < 0.001; ES = 0.67; 95% CI [0.07, 0.02]). However, when comparing the first maximum peak in velocity from pre- with the first maximum peak in velocity in the slowest time point (post0), we found that in the slowest time point, the first maximum peak was smaller (lower velocities were recorded prior to the initiation of the sticking region) (*p* = 0.004; ES = 0.64; 95% CI [−0.012, −0.063]). Additionally, the ascending phase velocity until the first maximum peak in the fastest time point was higher than in the post0 time point (*p* < 0.0001; ES = 1.22; 95% CI [0.112, 0.053]) (Figure 2B).

**Figure 3:**
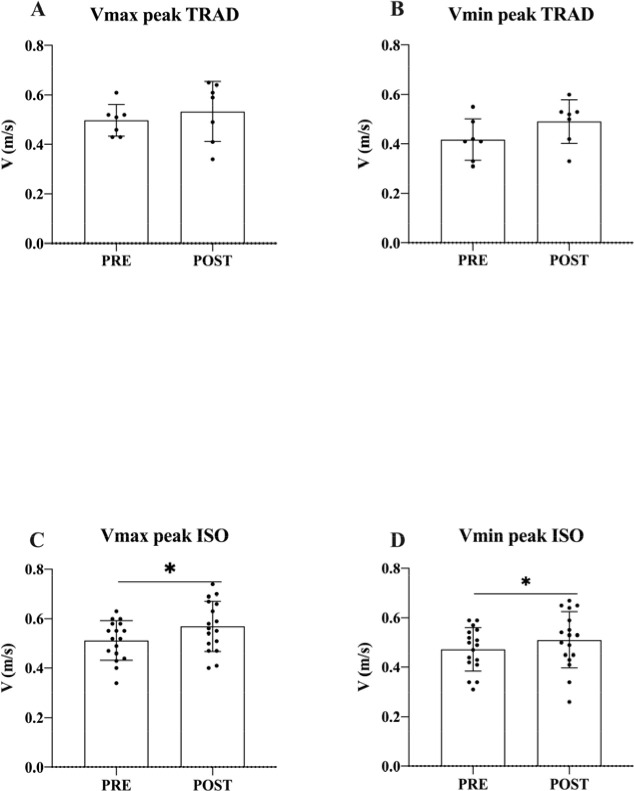
(A) First peak in the velocity of the load (V_max_) in pre- and the fastest post-conditioning lift in TRAD. (B) First local minimum velocity peak (V_min_) in pre- and the fastest post-conditioning lift in TRAD. (C) V_max peak_ in pre- and the fastest post-conditioning lift in ISO. (D) V_min peak_ in pre- and the fastest post-conditioning lift in ISO. * p < 0.05

We compared V_max peak_ and V_min peak_ from pre- to the fastest post-conditioning lift of each participant for whom either the TRAD or the ISO experimental condition was effective. The TRAD experimental condition showed no improvements in V_max_ _peak_ (*p* = 0.457; ES = 0.37; 95% CI [−0.074, 0.146]), nor in V_min peak_ (*p* = 0.125; ES = 0.85; 95% CI [−0.0271, 0.173]), while the ISO experimental condition showed improvements in both V_max peak_ (*p* = 0.005; ES = 0.71; 95% CI [0.02, 0.093]) and V_min peak_ (*p* = 0.025; ES = 0.38; 95% CI [0.006, 0.072]) (Figures 3A–D).

### Mean Velocity Changes and RPE

**Figure 4:**
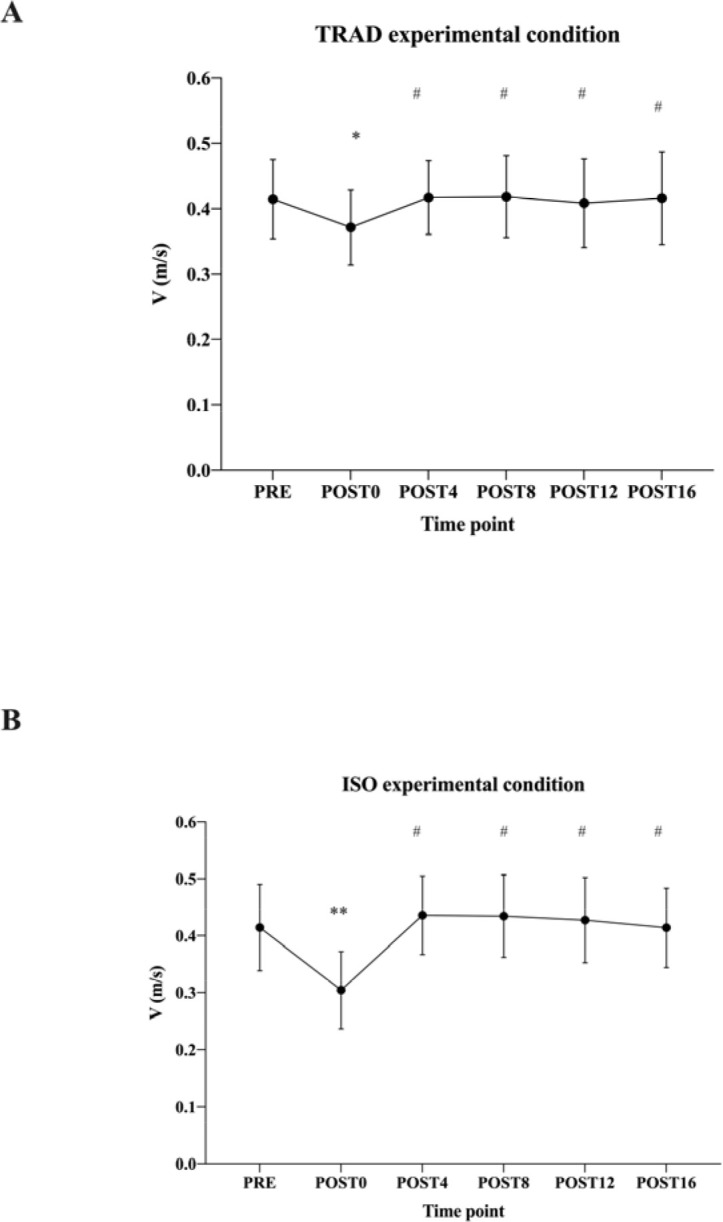
Lifting velocities pre and post 0, 4, 8, 12 and 16 minutes. (A) Mean TRAD post-activation performance enhancement experimental session values. (B) Mean ISO post-activation performance enhancement experimental session. Significantly different from pre * (p < 0.05) or ** (p < 0.001) and # post0 (p < 0.001)

When comparing the mean propulsive velocity between pre- and all post-conditioning lifts, we found that post0 was slower than pre- in both TRAD (*p* = 0.002; ES = 0.74; 95% CI [−0.0136, −0.073]) (Figure 4A) and ISO experimental conditions (*p* < 0.001; ES = 1.53; 95% CI [−0.067, −0.153]) (Figure 4B). The TRAD protocol reported an average RPE (rate of perceived exertion) of 7.3 ± 0.7, while the ISO protocol reported an average RPE of 7.0 ± 1.0. None of the post-measurements reached statistical significance compared to pre-conditioning values. When comparing the same time points across experimental conditions (ISO vs. TRAD), there was only a significant result in post0, where the ISO condition induced slower performance than the TRAD condition (0.37 m•s^-1^ vs. 0.3 m•s^-1^; *p* = 0.01; ES = 1.07; 95% CI [0.01219, 0.01217]).

## Discussion

**Figure 5:**
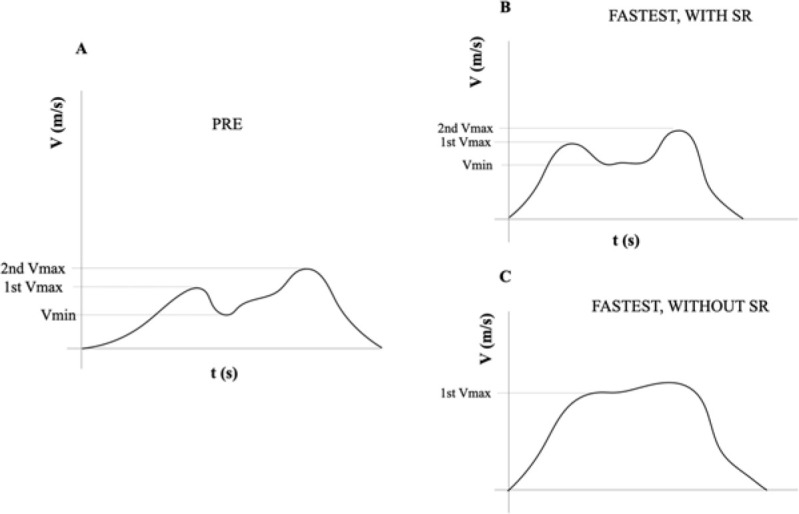
(A) Illustration of a typical Sticking Region velocity-time profile, which could be observed in pre-conditioning lifts. (B) Illustration of a Sticking Region velocity-time profile post-conditioning, with enhanced the first peak in the velocity of the load (V_max_) and its first local minimum peak thereafter (V_min_). (C) Illustration of a post-conditioning lift where the impulse prior to the initiation of the Sticking Region is augmented to the point that no velocity loss occurs and, thus, V_min_ disappears.

The findings of this study support one of our two hypotheses, i.e., the kinematics and the characteristics of the sticking region change considerably (Figure 5 A–C) after the ISO conditioning protocol, however, mean propulsive velocity remains unchanged.

The main finding of this study was that an isometric PAPE protocol improved sticking region kinematics in the first part of the range of motion of the medium grip bench press. This improvement is evident in the first period of the ascending phase, prior to the sticking region, which helps the lifter to overcome that sticking region (Kompf and Arandjelović, 2016). The main enhancement is that the first maximum velocity peak is greater after the ISO conditioning protocol (Figure 3C), which provides the lifter with a greater impulse to overcome the sticking region. Also, the minimum velocity is higher after the ISO conditioning protocol (Figure 3D). The augmented first maximum and minimum velocity peaks result in a change in the velocity-time profile of the lift (Figure 5 A–C). This change in the velocity-time profile of the lift and the sticking region (i.e., less velocity loss from maximum to minimum velocity points) is due to the greater impulse that the lifter has achieved prior to the sticking region. The improvement in the first part of the lift can provide the lifter with enough impulse to avoid excessive velocity loss from V_max peak_ to V_min peak_, making the sticking region imperceptible (Figure 5C).

The ISO conditioning protocol improved performance in more participants (85%) than the traditional conditioning protocol (33.33%), which could be related to the interaction between stimuli and fatigue (Chiu and Barnes, 2003). Recruitment of type II fibers is needed to achieve PAPE, which is the result of a correct selection of intensity (Garbisu-Hualde and Santos-Concejero, 2021). The TRAD conditioning protocol implied a higher RPE than the ISO conditioning protocol (ES = 0.36). Nonetheless, in both conditioning protocols, participants reported an RPE of 7, which is in line with previous research (Garbisu-Hualde and Santos-Concejero, 2021). The ISO conditioning protocol includes 1-s rest intervals, which via the reduction in inorganic phosphate accumulation could help reduce excessive fatigue (Skurvydas et al., 2019).

It is worth mentioning that the ISO conditioning protocol produced a greater decrease in performance immediately post conditioning (post0) compared to the TRAD protocol. This could be due to the total time under tension, which is greater in the ISO conditioning protocol (15 s in total). However, in contrast to the TRAD conditioning protocol, improvements in velocity until the initiation of the sticking region were found in the ISO conditioning protocol, which suggests that isometric contractions produce less fatigue than dynamic contractions, or that subjects recover from the produced fatigue faster (Lum and Barbosa, 2019). This lower cumulative fatigue may be due to the lower consumption of ATP in lengthened and isometric contractions compared to shortening contractions (Beltman et al., 2004). Regarding the neural factors limiting maximal force production, it is widely accepted that motor unit recruitment strategies play a key role (Farina and Negro, 2015). The origin of this central fatigue could be at the spinal level, due to inhibitory intramuscular afferents (i.e., group Ia and II muscle afferents) and recurrent inhibition by Renshaw cells (Gandevia, 2001).

We must acknowledge some limitations. Considering that a linear encoder provides the mean propulsive velocity (Pareja-Blanco et al., 2017) and that the minimum mean propulsive velocity for a successful lift in the bench press has been calculated (González-Badillo et al., 2011), more velocity implies more distance from that mean minimum propulsive velocity, and thus, furthers subjects failure. Unfortunately, changes in magnitude (e.g., from 0.27 m•s^-1^ to 0.29 m•s^-1^) were so small that statistical significance was not reached. Nevertheless, when comparing instantaneous velocities, magnitude changes were greater (e.g., from 0.26 m•s^-1^ to 0.34 m•s^-1^). Also, the selected intensity for the control lift (85% 1RM) was high. Even if notorious mean propulsive velocity changes are hard to see at those intensities, 85% 1RM was chosen for two main reasons: (i) higher similarity to real strength training or competition, and (ii) this is the minimum intensity needed to record a sticking region (Tillaar et al., 2014), which was one of the intentions of the study. Finally, it is worth mentioning that this study does not include a classic control group with no conditioning activity carried out. These limitations imply that conclusions of this study should be interpreted with caution.

## Conclusions

In conclusion, the results of this study suggest that short lasting maximal voluntary isometric contractions (15 x 1-s MVICs interspersed with 1-s rest intervals) result in better pre-sticking region kinematics, which could lead to reduced RPEs reported by athletes. It could be very useful when a rapid potentiation is needed, when athletes have not much time between the warm-up and the competition lift or when athletes feel insecure about managing heavy weights.
